# Tubal Origin of “Ovarian” Low-Grade Serous Carcinoma: A Gene Expression Profile Study

**DOI:** 10.1155/2019/8659754

**Published:** 2019-03-05

**Authors:** Yiying Wang, Shuhui Hong, Jingyi Mu, Yue Wang, Jayanthi Lea, Beihua Kong, Wenxin Zheng

**Affiliations:** ^1^Department of Obstetrics and Gynecology, Henan Provincial People's Hospital, Zhengzhou, China; ^2^Department of Obstetrics and Gynecology, People's Hospital of Henan University, Kaifeng, China; ^3^Department of Gynecology, Qianfoshan Hospital of Shandong University, Ji'nan, China; ^4^Department of Obstetrics and Gynecology, University of Texas Southwestern Medical Center, Dallas, TX, USA; ^5^Harold C Simmons Comprehensive Cancer Center at University of Texas Southwestern Medical Center, Dallas, TX, USA; ^6^Department of Obstetrics and Gynecology, Qilu Hospital, Shandong University, China; ^7^Department of Pathology, University of Texas Southwestern Medical Center, Dallas, TX, USA

## Abstract

**Objective:**

Ovarian low-grade serous carcinomas are thought to evolve in a stepwise fashion from ovarian epithelial inclusions, serous cystadenomas, and serous borderline tumors. Our previous study with clinicopathological approach showed that the majority ovarian epithelial inclusions are derived from the fallopian tubal epithelia rather than from ovarian surface epithelia. This study was designed to gain further insight into the cellular origin of ovarian low-grade serous carcinomas by differential gene expression profiling studies.

**Methods:**

Gene expression profiles were studied in 43 samples including 11 ovarian low-grade serous carcinomas, 7 serous borderline tumors, 6 serous cystadenomas, 6 ovarian epithelial inclusions, 7 fallopian tubal epithelia, and 6 ovarian surface epithelia. Comprehensive analyses with hierarchical clustering, Rank-sum analysis and Pearson correlation tests were performed. Final validation was done on selected genes and corresponding proteins.

**Results:**

The gene expression profiles distinguished ovarian low-grade serous carcinomas from ovarian surface epithelia, but not from fallopian tubal epithelia cells. Hierarchical clustering analysis showed ovarian serous tumors and ovarian epithelial inclusions were clustered closely in a branch, but separated from ovarian surface epithelia. The results were further validated by selected proteins of OVGP1, WT-1, and FOM3, which were highly expressed in the samples of the fallopian tube, ovarian epithelial inclusions, and ovarian serous tumors, but not in ovarian surface epithelia. The reverse was true for the protein expression patterns of ARX and FNC1.

**Conclusions:**

This study provides evidence in a molecular level that ovarian low-grade serous carcinomas likely originate from the fallopian tube rather than from ovarian surface epithelia. Similar gene expression profiles among fallopian tube, ovarian epithelial inclusions, and serous tumors further support that ovarian low-grade serous carcinomas develop in a stepwise fashion. Such findings may have a significant implication for “ovarian” cancer-prevention strategies.

## 1. Introduction

Ovarian cancer is a leading cause of cancer deaths among women. Among all types of ovarian cancers, ovarian epithelial cancers (OEC), particularly those with serous histology, are responsible for the majority of cancer related mortality [1]. Serous carcinomas of the ovary are divided into high-grade serous carcinomas (HGSC) and low-grade serous carcinomas (LGSC)[2]. This categorization arises from the difference seen with regard to epidemiological and genetic risk factors, precursor lesions, patterns of spreading, molecular events during oncogenesis, response to chemotherapy, and prognosis [3–5]. The cell of origin of ovarian serous cancers was thought to be derived from ovarian surface epithelia (OSE)[6, 7]. There are high-grade and low-grade serous cancers within the ovarian serous cancer category. In the last decade, multiple morphologic, epidemiologic, and molecular observations have shown that the majority of HGSCs are likely derived from the fallopian tube epithelia (FTE) rather than from the OSE [8–18]. Furthermore, gene expression profiles and patterns of ovarian HGSC are more similar to those of normal FTE other than OSE [19, 20]. In contrast to the tubal origin of HGSC, the cellular origin of LGSC remains controversial. In general, it is thought that LGSC develops in a stepwise fashion from ovarian epithelial inclusions, benign cystadenomas, and serous borderline tumors [2]. Kurman et al. in 2011 proposed that papillary hyperplasia of the fallopian tube could be the precursor lesions of ovarian serous borderline tumor based on limited experience of pure morphologic observation [21]. However, this observation was not supported by the findings of recent studies [22, 23], emphasizing the need to evaluate the pathogenetic association between papillary hyperplasia of the fallopian tube and borderline tumors. Meanwhile, there were two experimental based publications that addressed the cellular source of serous borderline tumor and LGSC, respectively. A study by Laury et al. explored the cell types of the fallopian tube with a biomarker PAX2 to compare the differentiation characteristics of ovarian serous borderline tumors and concluded that fallopian tube secretory cells are likely the source of serous borderline tumor [24]. The other study was from our own group with a different experimental approach [25]. We performed morphological and immunophenotypic evaluations of LGSC and its precursor OEI, serous cystadenoma, and serous borderline tumors to compare with the patterns of expression of the fallopian tube and OSE. We showed that LGSC is likely derived from tubal secretory cells through a secretory cell expansion process [25]. However, further genetic studies are needed to address the cell of origin of LGSC. The current study with gene expression profiling addresses two aims: (1) confirming if LGSC comes from FTE and (2) showing molecular evidence for a stepwise development fashion of LGSC from OEI, benign serous cystadenoma, and serous borderline tumor (SBT). The study may also uncover novel pathways that could be involved in the genesis of LGSC.

## 2. Materials and Methods

### 2.1. Tissue Specimens

Forty-three flash-frozen ovarian and tubal samples which were derived from 6 study groups including LGSC (n=11), SBT (n=7), serous cystadenoma (n=6), OEI (n=6), distal FTE (n=7), and OSE (n=6) specimens were collected. These samples (from year 2013 to year 2016) were obtained from three hospitals including one from China (Henan Provincial Peoples' Hospital) and two from USA (University of Arizona and University of Texas Southwestern Medical Center). All tumors were classified by using standard International Federation of Gynecology and Obstetrics criteria and confirmed by a gynecologic pathologist (WZ). Tubal epithelia were obtained by using the brushing procedure described recently by our group [26]. Normal ovarian epithelial samples were obtained by brushing the ovarian surface. Since ovarian surface epithelia may contain tubal epithelial cells [25], we made sure that only brushings containing at least 95% calretinin positive cells were used for gene expression analysis. OEI and serous cystadenoma samples were obtained by laser capture microdissection (LCM) as described previously [27]. Samples of SBT and LGSC were obtained by manual dissection under the microscope since these tumors contained abundant tumor cells. Collected samples were immediately suspended and frozen in RLT buffer (Qiagen, Valencia, CA).

Patients ranged in age from 28 to 57 years, with a median age of 45 years. Patients were all premenopausal except 3 that were postmenopausal. Patients with LGSC represented stage 1 (n=8), stage 2 (n=1), and stage 3 (n=2), and patients with SBT were stage 1 (n=6) and stage 3 (n=1). No patient studied received hormonal usage or treatment within the 6 months before surgical resection. All samples obtained during the proliferative phase of the ovarian cycle account for potential hormonal effects on gene expression. The study was approved by institutional review board of all institutions.

### 2.2. RNA Extraction, Linear Amplification, and Hybridization to GeneChip Arrays

RNA extraction including quality and quantity analysis, reverse transcription, linear amplification, and gene chip array analysis were done as previously described [28] and individual gene expression values were determined by using dChip perfect match-only model [29].

### 2.3. Clustering Study, Pearson Correlation Data Analysis, and Gene Profile Ontology Analysis

Expression array analyzed data were contrasted by the above 6 groups of tissue samples to see which stratifications produced the most pervasive differences. Setting a univariate* P* cutoff of 0.005, we identified 410 genes from 63,000 probe sets, suggesting a minimum false discovery rate (FDR). Hierarchical clustering using rank correlation with complete linkage was then applied to the matrix of the ranks of these genes crossing all samples. Based on the 410 differentially expressed genes, unsupervised hierarchical clustering was conducted. To further identify the potential origin of LGSC, we performed rank-sum analysis and Pearson correlation test using the 410 differentially expressed genes. The expression levels of each gene were ranked across samples of LGSC and other serous samples in descending order. The ranks are then summed over all samples of each group and correlation coefficient was calculated between LGSC and other groups using Pearson correlation analysis. In a nonparametric test, Kruskal-Wallis, contrasts were done both with and without inclusion of the normal tubal and ovarian pools. To assess the relative strengths of the group separation, we first identified the genes showing the greatest ability to differentiate 6 groups using Kruskal-Wallis tests.

Statistical analysis for testing differential expression carried out the DESeq R package (1.34.1, Bioconductor). DESeq provides statistical routines for testing differential expression by use of the negative binomial distribution. The resulting P values were adjusted using Benjamini and Hochberg's approach for controlling the false discovery rate. Genes with an adjusted* P* value <0.05 found by DESeq were assigned as differentially expressed. Differentially expressed genes were analyzed by gene ontology enrichment analysis.

### 2.4. Validation Studies with RT-PCR and Immunohistochemistry

Based on the gene expression profiling and gene ontology analysis, we selected 5 differentially expressed genes (OVGP1, WT-1, FOM3, ARX, and FNC1) between FTE and OSE samples for validation by quantitative RT-PCR. Oligonucleotide primers for each gene are presented in Table 1. GAPDH was used as an endogenous control. The comparative threshold cycle method was used to calculate relative quantitation among all sample groups. All experiments were performed in triplicate for both target and reference genes.

The protein expression levels of the above 5 genes were also investigated in tubal and ovarian tissue samples by immunohistochemistry (IHC). A total of 177 formalin-fixed paraffin tissue samples (different from patients who contributing samples for gene profile study) were tested by routine IHC. These included fallopian tubes including tubal fimbria (n=18), OSE (n=12), LGSC (n=28), SBT (n=35), serous cystadenoma (n=16), and OEI (n=68). The IHC method and evaluation criteria were described previously [30, 31]. Statistical analysis was done using ANOVA followed by least significant difference (*P* < 0.05). Comparison of unpaired proportions was by Fisher's exact test (*P* < 0.05).

## 3. Results

### 3.1. Tubal Epithelia, but Not Ovarian Surface Epithelia, Exhibited Indistinguishable Gene Expression Profiles of Ovarian Low-Grade Serous Carcinoma

**Figure 1:**
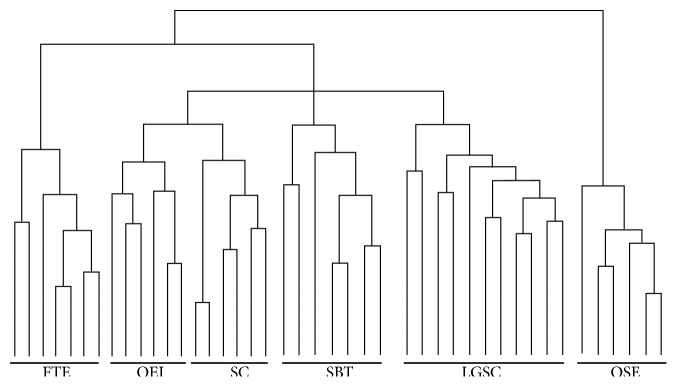
*Identification of differentially expressed genes in examined tissue samples*. Dendrograms produced by unsupervised hierarchical clustering according to differentially expressed genes, revealing the overall similarity of FTE and OEI and serous tumor samples. FTE samples are more closely resembling LGSCs compared with OSE samples. FTE, fallopian tube epithelia; OEI, ovarian epithelial inclusions; SC, serous cystadenoma; SBT, serous borderline tumor; LGSC, low-grade serous carcinoma; OSE, ovarian surface epithelia.

A total of 5956 differentially expressed genes were identified among the 6 groups of samples studied. Unsupervised hierarchical (agglomerative) clustering of the individual LGSC samples after robust multiarray average normalization showed that the samples of FTE and LGSC had closely related global gene expression profiles. In addition, profiles of OEI, serous cystadenoma, and SBT also clustered closely with that of FTE. In contrast, no clustering association was found from the samples of LGSC, SBT, serous cystadenoma, and OEI compared with the samples of OSE (Figure 1). To examine this relationship more closely, an unsupervised partitive clustering method, binary tree-structured vector quantization, was used. This method iteratively partitioned the 24 cases (11 LGSC, 7 FTE, and 6 OSE) by a* k*-means algorithm (*k*=2), considering the partitioning of all probe set responses by a self-organizing map algorithm. This method clearly revealed that samples partitioned together irrespective of presumed origin or known gene alteration status. Two-class paired significance microarray analysis of the two FTE and ovarian LGSC from the same patients revealed no differentially expressed genes at a minimum FDR of 30%, as did two-class unpaired analysis of the remaining LGSC specimens at an FDR of 18%. Therefore, the results indicate that LGSC have similar molecular profiles of the fallopian tube.

### 3.2. Gene Expression Profiles Were Similar among Samples of Ovarian Epithelial Inclusions, Serous Cystadenoma, Serous Borderline Tumor, and Low-Grade Serous Carcinoma

Among the above 4 groups (OEI, serous cystadenoma, SBT, and LGSC) of the samples, we used unsupervised hierarchical cluster method to analyze differentially expressed genes to determine the overall similarity among OEI, serous cystadenoma, SBT, and LGSC. Although similar, identical results were not obtained using the two clustering methods. This is likely due to the different disease development steps of the low-grade serous carcinogenesis. Again, the expression profiles of all 4 groups resembled significantly the FTE samples, but dramatically different from the OSEs. The data is summarized in the corresponding dendrogram (Figure 1). Rank-sum analysis and Pearson correlation test were applied to further determine the correlation between LGSC and FTE and OSE groups. A significantly greater correlation coefficient was found between LGSC and FTE, but not OSE (data not shown).

### 3.3. Differential Expression of Genes in the Spectrum of Serous Carcinogenesis

**Figure 2:**
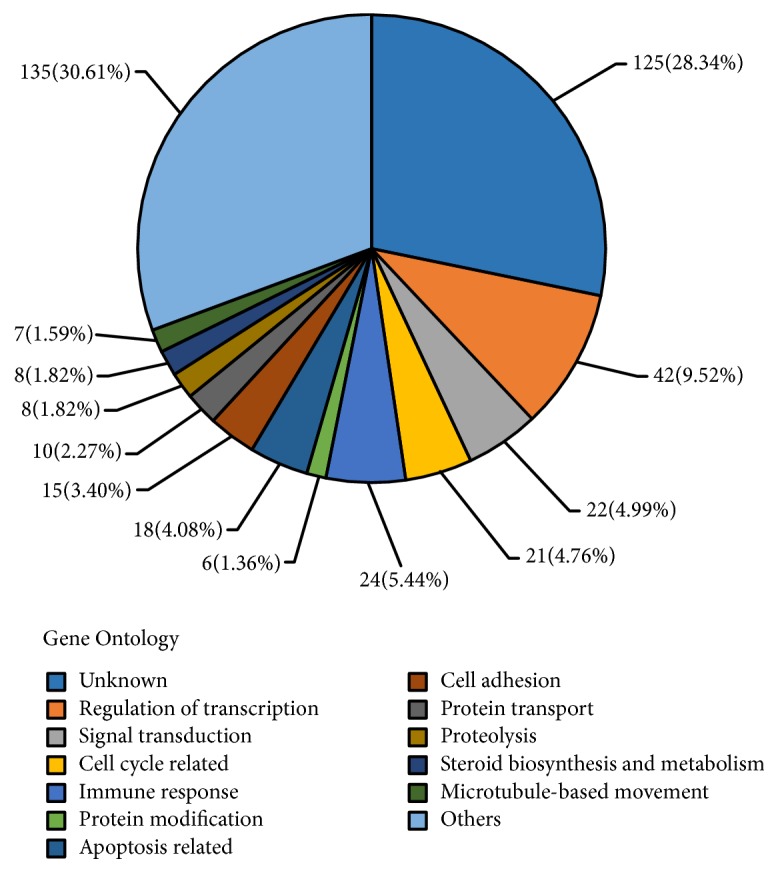
*Gene ontology showing overall differentially expressed genes.* Venn diagram was used to compare probe sets significantly altered in FTE samples to those differentially expressed between LGSC and FTE specimens at a false discovery rate of 4.1 (see results for details).

Hierarchical clustering analysis using grouped data revealed that FTE samples clustered with samples of OEI, serous cystadenoma, SBT, and LGSC rather than normal OSE, demonstrating that gene expression altered in mutation carriers is consistent with changes that have occurred in the process of LGSC development. These changes likely reflect and contribute to the overall increased risk for malignant transformation. By using two-class unpaired analysis, we identified 218 probe sets with decreased expression and 468 with increased expression in the 10 LGSC samples relative to the FTE samples that were more similar to normal controls at an FDR of 4.1%. Gene ontology analysis revealed that many differentially expressed probe sets correspond to genes with known roles in the processes of transcriptional regulation, cell cycle control, ubiquitin cycle, and others involved in tumor initiation and progression in general (apoptosis, cell adhesion, and cell motility) (Figure 2). In addition, 121 of 218 (55%) of the probe sets with decreased expression and 127 of 468 (27%) with increased expression overlapped in the same direction as those differentially expressed between OEI and LGSC samples as a group at the same FDR (4.1%), further supporting the idea that some of these downregulated genes, may represent potential early events in low-grade serous carcinogenesis.

### 3.4. Validation of the Gene Expression Findings by Quantitative RT-PCR and Immunohistochemistry

**Figure 3:**
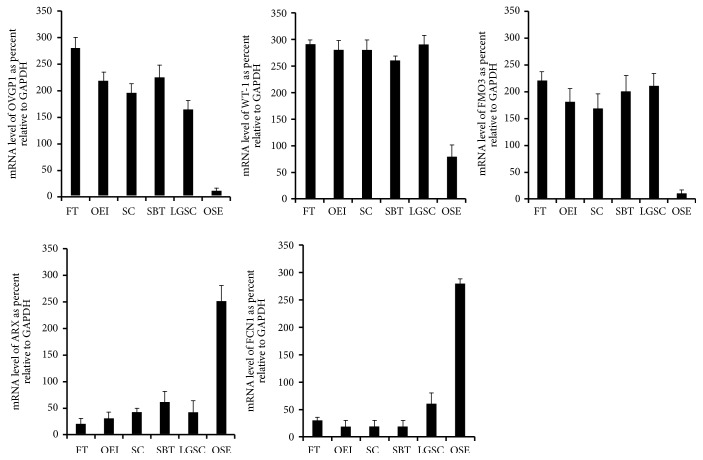
*Validation of differentially expressed genes by RT-PCR.* Gene expression determined by microarray analysis and quantitative RT-PCR for genes up- and downregulated in the fallopian tube and ovarian samples (OVGP1, WT-1, FMO3, ARX, and FCN1). Compared to OSE samples, the gene expression level of OVGP1(A), WT-1(B), and FMO3(C) was significantly higher in samples of FTE, OEI, SC, SBT, and LGSC (*p *< 0.0001), while no statistical differences were detected among the samples of FT, OEI, SC, SBT, and LGSC. Compared to FT, OEI, SC, SBT, and LGSC samples, the expression level of ARX(D) and FCN1(E) was significantly higher in OSEs (*p *< 0.0001), while no statistical differences were detected among the samples of FT, OEI, SC, SBT, and LGSC. FTE, fallopian tube epithelia; OEI, ovarian epithelial inclusions; SC, serous cystadenoma; SBT, serous borderline tumor; LGSC, low-grade serous carcinoma; OSE, ovarian surface epithelia.

To verify the results from the gene expression profiles, we selected the most significantly differentially expressed genes between FTE and OSE by RT-PCR. The following candidate genes were selected: OVGP1, WT-1, FMO3, ARX, and FCN1. The first three genes showed high levels of expression in ovarian serous tumors including the putative precursor OEIs and FTE, while the later 2 genes were more expressed in OSE. We used these genes tested additional freshly frozen samples including 8 FTEs, 10 ovaries with at least some OSE, 5 serous cystadenomas, 6 SBT, and 10 LGSCs. The results were compatible with the findings of the gene expression profiles. Representative pictures are presented in Figure 3.

**Figure 4:**
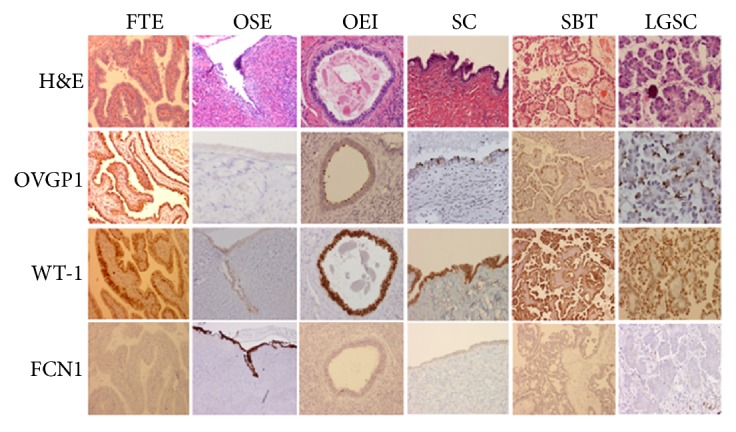
*Validation of differentially expressed proteins by IHC.* The top panel represents H&E stains, while the second to fourth panels represent immunohistochemical stains with the antibodies against OVGP1, WT-1, and FNC1, respectively. The findings are correlated with the gene expression profiles. OVGP1 and WT-1 are positively expressed in FT, OEI, SC, SBT, and LGSC, but negative in OSE. In contrast, FNC1 is expressed only in OSE, not in other tested samples. Original magnification: 100X. FT, fallopian tube; OEI, ovarian epithelial inclusions; SC, serous cystadenoma; SBT, serous borderline tumor; LGSC, low-grade serous carcinoma; OSE, ovarian surface epithelia.

In addition to the freshly frozen samples, we tested the corresponding formalin-fixed tissue samples by using immunohistochemistry. Antibodies against OVGP1, WT-1, and FMO3 showed strong expression in samples of FTE, OEI, and serous tumors including LGSC, while largely negative in OSE. In contrast, antibodies against ARX and FCN1 showed the opposite results. The detailed scores of individual biomarkers obtained by immunohistochemistry are listed in Table 2 and representative staining results are presented in Figure 4.

## 4. Discussion

The fallopian tube as the origin of ovarian HGSC has caused a paradigm shift in clinical approach to ovarian cancer [32]. The expanding acceptance of this concept has led to consideration of salpingectomy as a possible preventive method for ovarian cancer [33]. Although HGSC is responsible for approximately 70% of the ovarian epithelial cancer related mortality and morbidity, ovarian LGSC still plays a role in cancer related death. Further understanding the cell of origin and biology of non-HGSC including those LGSCs may contribute ideas for new prevention strategies, improve early detection, and test novel therapies.

Based on current understanding, LGSC likely develops in a stepwise fashion from OEI to serous cystadenoma and then to serous borderline tumor [2]. Prior studies that have supported this model by showing (1) serous borderline tumors and in adjacent serous cystadenoma epithelia are present similar mutations of KRAS and BRAF genes [34]; (2) OEIs from ovaries with serous borderline tumors have higher levels of epithelial cell aneusomy than that of OEIs from ovaries with nonneoplastic disease [35]; and (3) a strong relationship between LGSC and serous borderline tumors has been reported previously [36]. Due to similar epithelial linings, OEIs are thought to be the precursors of majority of serous cystadenomas and the diagnostic criteria for differentiating OEI and serous cystadenoma is based on the 1cm size threshold [37]. Based on this understanding, we previously evaluated the morphologic and immunophenotypic features of ovarian OEIs, OSE, and serous tumors including LGSC and distal tubal epithelium several years ago and concluded that the majority OEIs are derived from the fallopian tube [25]. However, that was a clinicopathologic study, which may not be enough to move the concept forward to guide clinical practice. In the current study, we used the genetic profiling technology to examine if we can identify molecular evidence to support our clinicopathologic observations. From historic perspective, it remains unclear where OEI is derived from fallopian tube or OSE. Therefore, both samples of fallopian tube and OSE were included in the study in addition to the samples from different steps of LGSC development. If our previous observation [25] holds, then we might expect significant differences of the gene expression profiles between fallopian and OSE when compared to the samples from OEI to ovarian LGSC, while a reasonable closer association of gene expression profiles should be present among serous cystadenoma, borderline tumor, and LGSC. To test this hypothesis, we first arrayed a panel of 7 normal tubal and 6 ovarian surface epithelial samples for comparison showing a dramatic difference of expression profiles. Next, by contrasting samples of OEI and 3 groups of ovarian serous tumors, we assembled a list of genes we would use for comparing the normal tubal or ovarian epithelia and checking for parallelism. There is one list of genes per group. A gene made it onto the LGSC list (for example) by having an average expression level in other groups that was (a) >2-fold greater or less than the average expression level in each of the 5 other groups and (*b*) >100 units greater (less) than the average expression level in each of the 5 other groups.

Our data, with a reasonably good sample size, confirm that gene expression profiles clearly distinguish LGSC from OSE, but not from FTE cells. Histology related changes in gene expression correlated with gene expression in FTE samples serous cystadenoma when normal OSE cells were used as a standard for comparison. Pathologists have noticed the resemblance of the epithelial cells of serous tumors including the OEI (endosalpingiosis) to fallopian tubal epithelia. The current study shows for the first time that genes expressed in the different steps of ovarian LGSC development (OEI, serous cystadenoma, SBT, and LGSC) are also concordantly expressed in the cell of origin that they resemble histologically.

According to the data obtained in this study, multiple genes were upregulated in normal fallopian tube as well as in the samples of OEI, serous cystadenoma, SBT, and LGSC. We verified three upregulated genes in the study: OVGP1, WT-1, and FMO3. OVGP1 encodes a protein that is secreted by nonciliated cells in the mammalian fallopian tube and appears to play a role in optimizing the microenvironment for oocyte maturation and transport, fertilization, and early embryonic development [38]. Due to its relatively specific expression in the fallopian tube, this protein has been used as an organ specific marker to examine if the fallopian tube is the cell of origin of ovarian epithelial cancers in transgenic mouse models [39, 40]. Interestingly, this tubal produced glycoprotein has been detected in the OEI and various ovarian epithelial tumors including SBTs [41]. The WT1 gene, located at chromosome 11p13, provides instructions for making a protein that is necessary for the development of the kidneys and gonads. Within these tissues, WT1 protein, a transcription factor, plays a role in cell growth, the process by which cells mature to perform cellular differentiation or apoptosis. Within pathology, WT-1 protein expression is considered as a serous differentiation marker since it is positively expressed in many ovarian serous tumors including LGSC. FMO3, flavin-containing monooxygenase 3 encoding microsomal flavin-containing monooxygenase, plays an important role during the oxidative metabolism of a variety of xenobiotics. Its expression is sex-independent in human and enriched in the adult liver. This gene and protein was also highly expressed in the fallopian tube in our previous study of tubal origin of ovarian endometriosis [30]. All these three genes and their corresponding proteins are highly expressed in the fallopian tube, but rarely or not expressed in the OSE samples. Meanwhile, these genes and proteins are also highly expressed in the samples of OEI, serous cystadenoma, SBT, and LGSC. Such findings are supportive of tubal origin of ovarian LGSC and it likely develops from OEI to serous cystadenoma and SBT in a stepwise fashion as observed morphologically. FMO3 is recently known to be expressed in serous tumors. All three markers may be considered as candidates for ovarian serous cancer early detection.

There are multiple significantly downregulated genes found in this study. Both ARX and FCN1 had a very low level of expression in samples of FTE, OEI, serous cystadenoma, SBT, and LGSC, but highly expressed in OSEs. The ARX gene, located at the short arm of X chromosome, is part of a larger family of homeobox genes, which act during early embryonic development to control the formation of many body structures including pancreas, testes, brain, and skeletal muscles [42]. ARX protein, as a transcription factor within the developing brain, is involved in neuron migration and communication [42]. LCN1 gene is located on the long arm of chromosome 9. The function of its encoding products, lipocalins, serves as an extracellular transporter by binding a variety of hydrophobic ligands and it may be related to the development of Sjogren's syndrome [43]. However, it is currently unknown for the functions of these genes and their protein products in the women's pelvis and their biologic roles when they highly expressed in the cells of OSE. However, these markers may be used in surgical pathology to help differential diagnosis of mesothelial lesions from disease of Mullerian origin.

## 5. Conclusions

This study provides a genetic profiling evidence that ovarian LGSC most likely originates from the fallopian tube rather than from ovarian surface epithelia. In conjunction with evidence in the published literature and our previous studies, we believe that the development of ovarian LGSC likely follows the following steps. First, the fallopian tubal epithelia, mostly the fimbriated end, becomes adherent to the ovarian surface. This process can be facilitated by chronic inflammation, ovulation, and nonovulation induced disruption of the ovarian surface, which are common activities in reproductive aged women [2]. The adherent tubal epithelia then have a chance of invagination into ovarian cortex to form OEI [25]. The acquisition of KRAS or BRAF and possibly other mutations including PAX-2 in OEIs and serous cystadenomas result in their gradual transformation to serous borderline tumors and ultimately LGSC [44–48]. Morphologically, the process of LGSC development is illustrated by the gradual increment of tubal secretory cells and loss of tubal ciliated cells, which may represent some genetic hits deregulating normal cellular differentiation [25]. The current findings, which further augment the important role of fallopian tube in ovarian cancer development, may have implications for the ovarian epithelial cancer-prevention strategies.

## Figures and Tables

**Table 1:** Sequence of primers used in this study.

Primer name	Primer sequence (5'-3')
WT-1 (F)	GGGGGAGGGTTGTGTTATAT
WT-1 (R)	CTCCTTACCCCAACTACCTAACTAC
OVGP1 (F)	ACGTCTTATGATGCGCTCCTT
OVGP1 (R)	TTATCTGCGGGTGTCCCAAG
FMO3 (F)	AATTCGGGCTGTGATATTGC
FMO3 (R)	TTGAGGAAGGTTCCAAATCG
ARX (F)	GTGCAAGGCTCCCCTAAGAG
ARX (R)	CGTTCTCGCGGTACGACTT
FCN1 (F)	GGGCAGTGCGGGTAATTCTC
FCN1 (R)	GAAGCATGACAGTCGGCGTA

**Table 2:** Immunohistochemistry scores of differentially expressed biomarkers.

	FTE	OEI	SC	SBT	LGSC	OSE
VGP1	280±20	218±17	196±16	226±21	164±18	12±5
WT-1	290±10	280±18	280±20	260±10	290±10	78±22
FMO3	220±18	180±26	168±28	200±30	210±24	10±8
ARX	20±10	30±12	40±10	60±20	40±24	250±30
FCN1	30±6	20±10	20±10	20±10	60±20	280±10

FTE, fallopian tubal epithelia; OEI, ovarian epithelial inclusions; SC, serous cystadenoma; SBT, serous borderline tumor; LGSC, low-grade serous carcinoma; OSE, ovarian surface epithelia.

Compared to OSE samples, the expression level of OVGP1, WT-1, and FMO3 was significantly higher in tissues of FTE, OEI, SC, SBT, and LGSC (p < 0.0001), while no statistical differences were detected among the samples of FTE, OEI, SC, SBT, and LGSC. Compared to FTE, OEI, SC, SBT, and LGSC samples, the expression level of ARX and FCN1 was significantly higher in OSEs (p < 0.0001), while no statistical differences were detected among the samples of FTE, OEI, SC, SBT, and LGSC.

## Data Availability

All data and materials for the project are either kept in PI (WZ) desktop or remained in the Department of Pathology, UT Southwestern Medical Center.
